# An Unusual Case of Lumen-Apposing Metal Stent–Related Splenic Capsule Hemorrhage

**DOI:** 10.1093/jcag/gwaa038

**Published:** 2020-12-14

**Authors:** Dalton R Budhram, Reza Nasirzadeh, Lawrence Hookey

**Affiliations:** 1 Department of Medicine, Queen’s University, Kingston, Ontario, Canada; 2 Department of Radiology, Queen’s university, Kingston, Ontario, Canada; 3 Gastrointestinal Diseases Research Unit, Department of Medicine, Queen’s University, Kingston, Ontario, Canada

**Figure 1.**
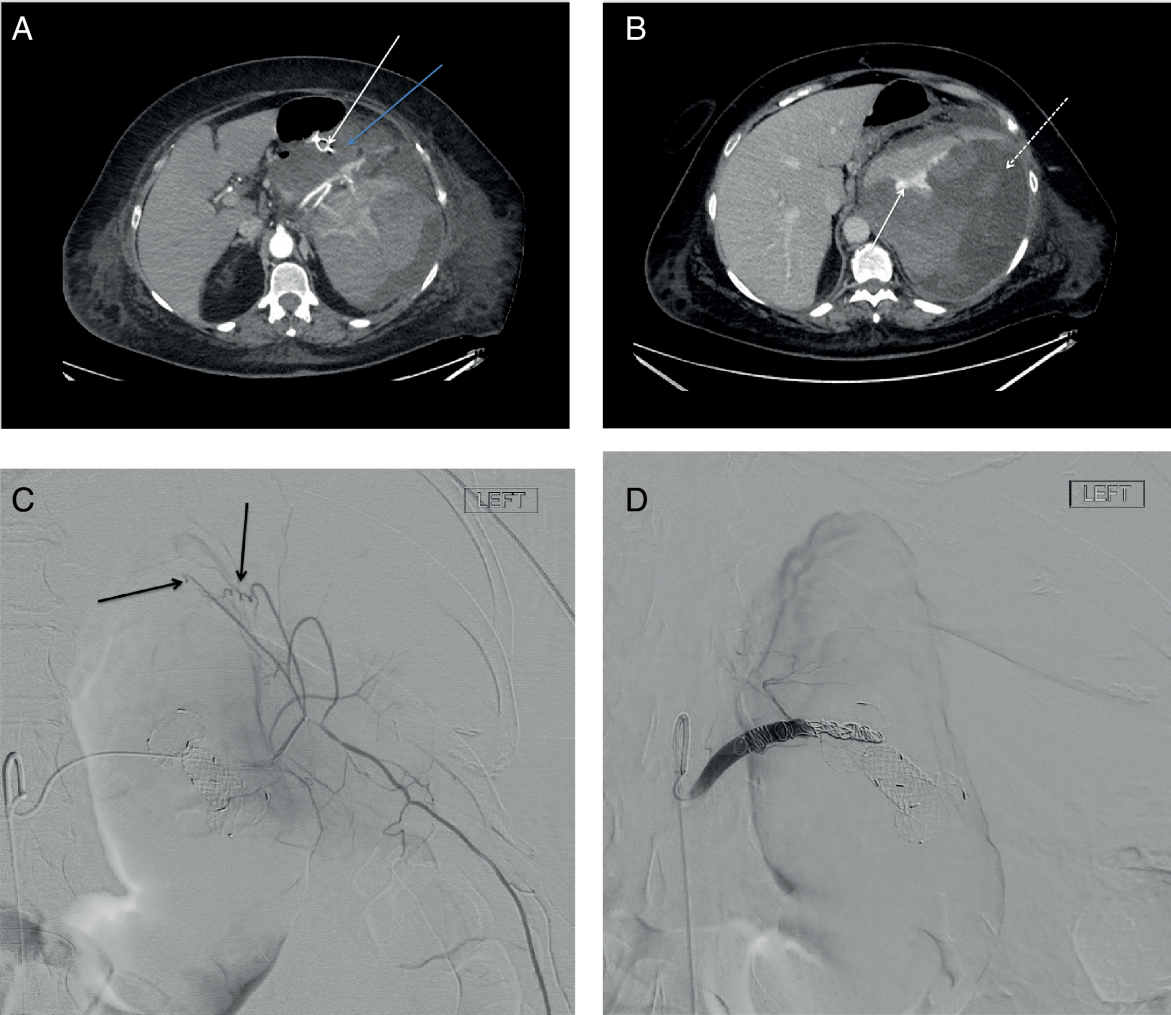
Radiographic images of pseudocyst drain and angiography of bleeding site. (A) Computerized tomography (CT) abdomen: blue arrow—multiple large fluid collections, largest 9.8 x 11.4 x 8.7 cm in the pancreas tail region; white arrow—4 cm x 12 mm lumen-apposing metal stent (Hanaro Stent, M. I. Tech, Korea) placed for drainage, (B) CT abdomen with contrast: solid arrow—fluid collection measuring 12 cm with the accumulation of extravasated contrast suggesting hemorrhagic transformation; dashed arrow—large splenic subcapsular/pericapsular hematoma, (C) arteriogram revealing abnormal distal capsular splenic branches with no active extravasation and (D) angiogram of the splenic artery showing proximal embolization with platinum coils.

A 54-year-old woman with acute pancreatitis 6 months ago presented with abdominal pain and vomiting in the past 2 weeks. A computerized tomography (CT) abdomen showed multiple large fluid collections, largest 9.8 × 11.4 × 8.7 cm in the pancreas tail region (Figure 1A, blue arrow). Using transgastric endoscopic ultrasound, the collection was visualized with some layering of solid material. A cystotome was used to puncture the collection and a 4 cm × 12 mm lumen-apposing metal stent (Hanaro stent, M. I. Tech, Korea) was placed for drainage (Figure 1A, white arrow).

The next morning her hemoglobin dropped by 31 g/L (from 83 to 52 g/L), but there was no evidence of hematemesis. She was transfused with two units of packed red blood cells and a CT with contrast was done to assess for bleeding. CT showed the fluid collection measuring at 12 cm with accumulation of extravasated contrast suggesting hemorrhagic transformation (Figure 1B, solid arrow) and large splenic subcapsular/pericapsular hematoma (Figure 1B, dashed arrow), displacing the spleen anteroinferiorly and measuring 12 × 17 cm.

An arteriogram revealed abnormal distal capsular splenic branches with no active extravasation (Figure 1C). The splenic artery was coiled proximally, which achieved good hemostasis and she recovered well (Figure 1D). A repeat CT 1 week later showed a smaller pseudocyst (8 × 7 × 4 cm) with evolution of the splenic hematoma. Three months later endoscopy was repeated, and the stent was removed without any issues or complications.

While several studies have reported adverse events relating to lumen apposing metal stent, this case documents the first splenic capsule hemorrhage associated with lumen apposing metal stent for draining pancreatic fluid collections.

